# Effectiveness of Different Preventive Programs in Cariogram Parameters of Young Adults at High Caries Risk

**DOI:** 10.1155/2017/7189270

**Published:** 2017-05-29

**Authors:** Said Karabekiroğlu, Nimet Ünlü

**Affiliations:** Department of Restorative Dentistry, Faculty of Dentistry, Necmettin Erbakan University, Konya, Turkey

## Abstract

**Objective:**

To evaluate the effectiveness of different preventive programs in young adults at high caries risk using Cariogram software.

**Methods:**

Sixty-six young adults with high caries risk were evaluated. Dental caries risk for all subjects was determined according to WHO criteria. Subjects were divided into three different preventive groups (control: OH, fluoride varnish: FV, and chlorhexidine varnish: CV). They were followed for 12 weeks (baseline: T0, 1 week: T1, 4 weeks: T2, and 12 weeks: T3). Plaque index, diet frequency, and salivary chairside tests (to record the flow rate, buffer capacity, and mutans streptococci and lactobacillus counts) were performed at each visit. Based on these data, ten caries-related variables were collected and inserted into the Cariogram software to calculate the predicted chance of avoiding caries for each subject.

**Results:**

Significant changes were obtained about the Cariogram parameters (diet, bacteria, susceptibility, circumstances, and Cariogram risk group). No significant differences were found between the three methods regarding mean Cariogram scores after 3 months (*p* > 0.05).

**Conclusions:**

The regular and effective short-term (three months) use of 1450 ppm fluoridated toothpaste, one visit application of fluoride, and chlorhexidine varnishes were effective for reducing caries risk in young adults, which can be clearly demonstrated using Cariogram software.

## 1. Introduction

Dental caries is still a major health problem worldwide with a multifactorial etiology, as it is due to the interaction of various factors including diet, the host's susceptibility, and the presence of microorganisms over a certain length of time [1]. Along with the dramatic decline in caries prevalence during the past 30 years in industrialized countries, the search for acceptable, accurate, and cost-effective strategies for identifying high-risk individuals has been intensified, and multiple risk factors and indicators have been proposed as targets [2]. It is therefore important to include assessment of caries risk in the development of targeted preventive measures [2]. Although many different models for predicting caries risk have been developed, none have proved very effective [3]. The risk profile is an important factor in decision-making processes to prevent and manage caries [4].

Numerous caries risk prediction and evaluation models have been developed. They are all designed to evaluate the caries risk in a patient or a population as accurately as possible, but none has predominated over the others [2–5]. Although etiological factors that have a role in the formation of caries are certainly presented, it is stated that the estimation of caries risk is not 100% successful. Estimating the individuals who will develop caries in the near future by determining the individual caries risk is reported to be very important for selecting the most suitable method to be used in the diagnosis, prevention, and treatment of caries [5]. The free-share software Cariogram was developed in an attempt to solve the problem that arises when applying these risk prediction methods, and it appears to evaluate patients more accurately than other risk prediction models [4–6].

The aims of Cariogram are to (a) identify those persons who will most probably develop caries and (b) provide these individuals with the appropriate preventive and treatment measures to arrest the disease [5, 6]. This program aims to demonstrate the multifactorial background of dental caries by illustrating the interaction of nine carious-related factors. Patients are scored on diet, plaque, caries experience, bacterial counts, and saliva secretion, and the results are shown as a pie chart risk profile [7]. The “Cariogram” has been used extensively in several countries and has demonstrated fairly high efficacy and good reliability [3–5]. The program also offers some recommendations to prevent the likelihood of caries in the near future. On the other hand, it is stated that Cariogram does not provide the number of caries that may occur in the future but rather dwells on the potential risk scenario [2].

Dental caries can be prevented by applying suitable measures; hence it is very important to identify those individuals who are most likely to develop dental caries through caries risk assessment and to provide them with the required preventive measures to interrupt the disease process [8]. There is a large body of evidence to support the use of topical fluorides (varnish, gel, toothpaste, and mouth rinse) and fissure sealants for caries prevention. Mutans streptococci (MS) have been shown to play a major role in the initiation of the caries process, and the use of antimicrobials, as an alternative to or in combination with fluoride (F), has also been explored [9]. Although a number of caries preventive measures, such as fluoride, chlorhexidine, and sealant applications, and professional prophylaxis, have proved efficacious in explanatory clinical trials, there is little information on their usefulness with high caries risk groups in everyday practice [10]. Despite these findings, further research is needed to explore the consequences of different preventive applications on caries-related plaque and salivary parameters and how this in turn may modulate individual caries risk [11].

However, to the best of our knowledge, no study has addressed the effects of different preventive programs on Cariogram parameters of high caries risk in young adults. Therefore, the aim of the present study was to assess consecutively the caries risk alterations of young adults at high caries risk following 12 weeks of three different caries preventive methods (1450 ppm fluoridated toothpaste, fluoride, and chlorhexidine varnish) using Cariogram.

## 2. Subjects and Methods

Ethical approval for this study was obtained from the Ethics Committee of the Dentistry School of the University of Selcuk (2012/10-22), and informed consent was obtained from all participants. A total of 246 (148 female and 98 male) young adults 18–25 years participated in this clinical study. Subjects were randomly selected in three different clinics during April 2012–September 2012.

### 2.1. Clinical Examination

Carious defects, fillings, and missing teeth were diagnosed, and DMFT (decayed, missing, and filled teeth index) scores were calculated according to WHO guidelines [12]. Clinical examination was made using a plane mouth mirror and blunt sickle probe with the aid of a dental chair light on dried teeth by one examiner (Said Karabekıroglu). Digital bitewing images were obtained using the same intraoral unit (Trophy CCX Digital Periapical X-Ray Machine, France) using number 2 Digora phosphor plates at 65 kV, 8 mA. After the plates were exposed, they were processed by Soredex Digora Optime, France. The clinical and radiographic data were recorded separately for each subject by the same examiner. All bitewing radiographs included the mesial surface of the first premolar and the distal surfaces of the second molar and no artifact, position, or processing errors. All proximal surfaces could be observed clearly in the bitewings.

### 2.2. High-Risk Group

The significant caries index (SiC) was calculated to select the one-third of the population with the highest caries scores. Therefore, 82 subjects were evaluated to be at high caries risk. The exclusion criteria for the patients were (1) systemic problem, (2) undergoing fixed orthodontic treatment, (3) smoking habits, (4) extreme plaque accumulation and periodontal problems, (5) no brushing habits, (6) taking antibiotics or any drugs in the last 3 months, and (7) regularly using fluoride mouthwash or other preventive methods. Sixteen subjects (systemic problems: 2, fixed orthodontic treatment: 2, smoking: 9, and taking drugs: 3) were excluded from study. A power analysis was established by G^*∗*^Power software (Ver. 3.0.10; Franz Faul, Universitat Kiel, Germany). A total sample of 66 subjects (22 per group) would give more than 80% power to detect significant differences with a 0.25 effect size between the three groups at a *p* = 0.05 significance level.

### 2.3. Study Design

The study was carried out over a period of 12 weeks, with a total of four visits: first visit (baseline: T0), second visit (1 week: T1), third visit (4 weeks: T2), and final visit (12 weeks: T3). The visits were scheduled at the same time of day for each individual. Prior to each visit, the volunteers were asked to avoid tooth brushing and all other oral hygiene measures 24 h in advance and not to eat or drink anything but water for 1 h before the visit.

### 2.4. Before Intervention

All teeth that had carious lesions were restored with resin composite or amalgam. Each patient was given the same instructions with respect to oral hygiene. Using a small mirror with which the subject could also see the teeth inside the mouth, any plaque and gingival margins were shown to the subject and the importance of cleaning was well emphasized to them. All patients received the usual home-care oral hygiene instructions and a packet with nonprescription fluoride toothpaste (1450 ppm of fluoride), a manual toothbrush, and dental floss. The use of sugar-free chewing gum after meals was recommended for everyone. Each patient was given the same diet advice, such as reduction of daily intake (the amount and frequency of consumption of sugars should be reduced, they showed avoid sugar-containing foods and drinks at bedtime, and added sugars should provide less than 10% of total energy in the diet or 60 g per person per day, whichever is the lesser). Potentially cariogenic foods and drinks (cakes and biscuits, sugar and chocolate confectionery, jams, preserves, honey, and sugared soft drinks) were described to the subjects. In addition, the results of the lactobacilli (LB) tests were shown and explained to the subjects at the first visit.

### 2.5. Intervention

Subjects were randomly allocated into three groups (*n* = 22 for each group): a control group (OH), fluoride varnish group (FV), and chlorhexidine varnish group (CV).

*OH*. Those in the control group were informed and encouraged to use the fluoride toothpaste (Colgate Total, 1450 ppm F, São Paulo, Brazil) three times a day during the 12 weeks after the first visit.

*FV*. The participants received topical application of a 5% sodium fluoride (22,600 mg/L F) varnish (Premier Dental, PA, USA). Firstly, the subjects' teeth were cleaned with a toothbrush. Secondly, excessive saliva in one or two quadrants of the mouth was removed by cotton rolls or by using an air syringe. It was not necessary to keep the tooth surface extremely dry because varnish could set in the presence of saliva. Thirdly, FV was applied to the tooth surfaces using a miniature cotton swab or brush, with the applicator dabbed repeatedly onto the tooth surface without contacting soft tissues. After a few minutes, a thin and clear layer was formed. Then the next quadrants were treated in the same manner. Patients were advised not to brush their teeth or chew food for at least 4 h after varnish application; during this time, soft food and liquid might be consumed. They were also told to use fluoridated dentifrices (1450 ppm F) for 12 weeks at home.

*CV*. The individuals received a 1% chlorhexidine diacetate and 1% thymol varnish (Cervitec®Plus, Ivoclar-Vivadent, Schaan, Liechtenstein) application once a time after baseline measurement. Cervitec Plus application procedures were as follows: the tooth surfaces were cleaned thoroughly, dried with an air syringe, and isolated with cotton rolls. Three drops of Cervitec Plus were poured into a Dappen dish. A thin coat of varnish was applied by means of a Vivadent applicator on all surfaces of the teeth. The varnish was dispersed with air and allowed to dry, and the cotton rolls were removed after 30 seconds. Subjects were instructed not to rinse, eat/drink, or brush for one hour. They were also told to use fluoridated dentifrices (1450 ppm F) for 12 weeks at home.

### 2.6. Saliva Sampling

Samples were collected in the morning between 9 and 12 a.m. under standardized conditions. Stimulated saliva was collected while chewing on a piece of paraffin wax for 5 minutes in a test tube graduated in milliliters, and the saliva secretion rate was expressed as milliliters per minute (mL/min). During collection, the patient was in an upright position. The buffer capacity of stimulated saliva was determined using the CRT Buffer® strip (Ivoclar-Vivadent, Schaan, Liechtenstein). After 5 min of reaction, a comparison was made with the colored chart provided by the manufacturer, and the buffer capacity was scored as low (pH below 4), medium (pH between 4.5 and 5.5), or high (pH over 6). The fresh saliva sample was then used to determine MS and LB counts. The CRT® Caries Risk Test (Ivoclar-Vivadent) was used to record the salivary counts of MS and LB. Both agar surfaces were wetted with saliva using a pipette without scratching the agar surface. The test vial was placed upright in the incubator (Ivoclar-Vivadent) and incubated at 37°C/99°F for 48 hours. After removal of the vial from the incubator, the densities of the MS and LB colonies were compared with the corresponding evaluation pictures in the enclosed model chart (0 = 0–<10^3^, 1 = 10^3^–10^4^, 2 = 10^4^–10^5^, and 3 =>10^5^ CFU/mL).

### 2.7. Creating a Risk Profile Using Cariogram

A caries risk profile of each individual was obtained at each of the four visits using Cariogram software [11]. For each subject, the following ten caries-related variables were put into the Cariogram software: (1) caries experience, (2) related diseases, (3) diet content, (4) diet frequency, (5) MS count, (6) amount of plaque, (7) fluoride program, (8) salivary buffer capacity, (9) saliva secretion rate, and (10) clinical examination. Based on the entered variables, the chance of avoiding caries in the future was calculated. Country/area was set at normal, and the group was high risk for all subjects. Owing to the high-risk level of the study group, DMFT was scored from 6 to 13 (DMFT: 6 = Score 1, DMFT: 7-8 = Score 2, DMFT: 9-10 = Score 2, and DMFT > 11 = Score 3). The plaque index of six teeth (16, 12, 24, 36, 32, and 44) was evaluated using Silness and Loe's scale [13]. The scoring of the salivary secretion rate was determined as follows: Score 0 = >1.1 mL/min, Score 1 = 0.9–1.1 mL/min, Score 2 = 0.5–0.9 mL/min, and Score 3 = <0.5 mL/min. Estimation of the dietary content was made using the salivary LB counts (as a measure of the cariogenic diet) [11]. Diet frequency was also evaluated: Score 0 = maximum 3 intakes/day, Score 1 = 4 or 5 intakes/day, Score 2 = 6 or 7 intakes/day, Score 3 = over 7 intakes/day (4). The fluoride variable was scored as 1 for each visit in the FV group, except for the baseline. For all individuals in the present study, the “clinical judgment” factor was given a score of one. Finally, the caries risk profile for each participant was obtained as a pie chart with five colored sectors, which showed the chance of avoiding caries as a percentage. According to these percentage values, all subjects were scored into three modified groups (high [H]: 0–30%, medium [M]: 31–60%, low [L]: 61–100%) from the highest to the lowest predicted risk group.

### 2.8. Statistical Analysis

The statistical analysis was processed with the SPSS 17.0 software system (SPSS Inc., Chicago, Illinois, USA). A *p* value of <0.05 was considered statistically significant. All microbiological analyses and “Cariogram” calculations were carried out coded. Descriptive statistics, including means, standard deviations, and frequencies (percentages), were calculated. The Cariogram variables included in the statistical analysis were (1) diet (dietary content and frequency), (2) bacteria (MS level and plaque index), (3) susceptibility (fluoride program, saliva buffer capacity, and saliva secretion rate), (4) circumstances (caries experience and related diseases), (5) the predicted chance of avoiding caries, and (6) Cariogram risk levels. Diet, bacteria, susceptibility, and circumstances data were analyzed for statistically significant differences using Kruskal-Wallis and Wilcoxon tests. A one-way analysis of variance (ANOVA) and Tukey HSD tests were used to evaluate differences between the means for actual chance of avoiding caries for each group. A Friedman test was used for differences in Cariogram risk levels.

## 3. Results

**Figure 1:**
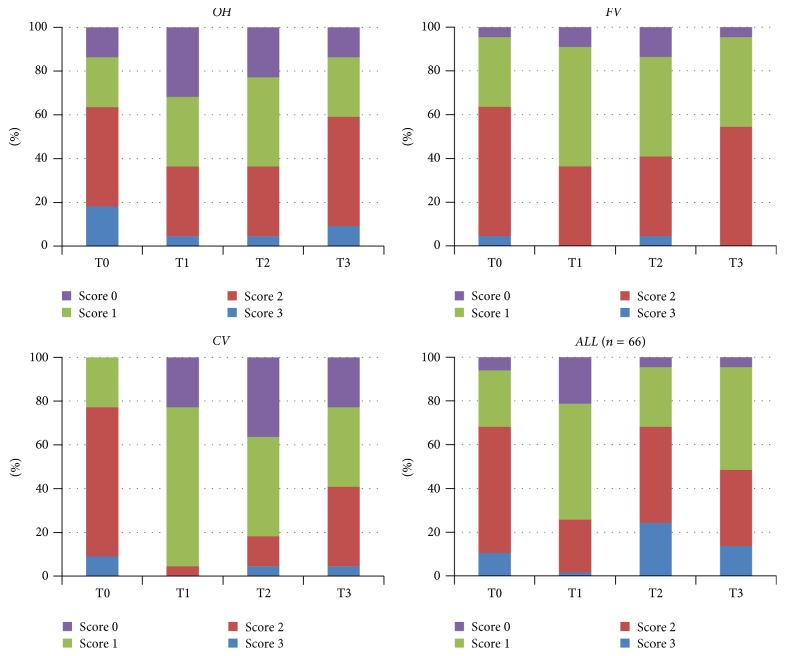
Sample distribution across the different visits (baseline and 1, 4, and 12 weeks) for salivary MS levels for all groups (*p* values between groups for different visits; T0: 0.305, T1: 0.03, T2: 0.07, and T3: 0.25).

**Figure 2:**
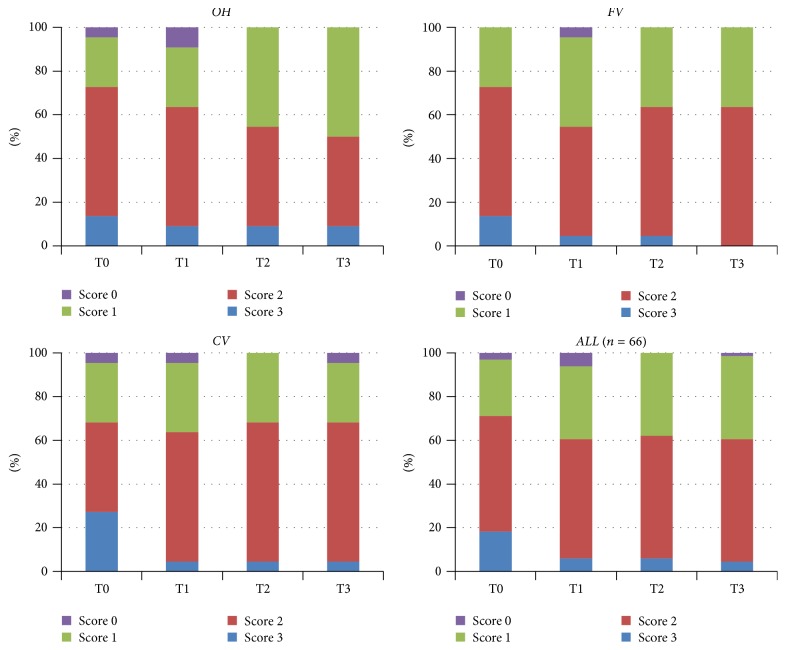
Sample distribution across the different visits (baseline and 1, 4, and 12 weeks) for salivary LB levels for all groups (*p* values between groups for different visits; T0: 0.684, T1: 1.000, T2: 0.607, and T3: 0.614).

**Figure 3:**
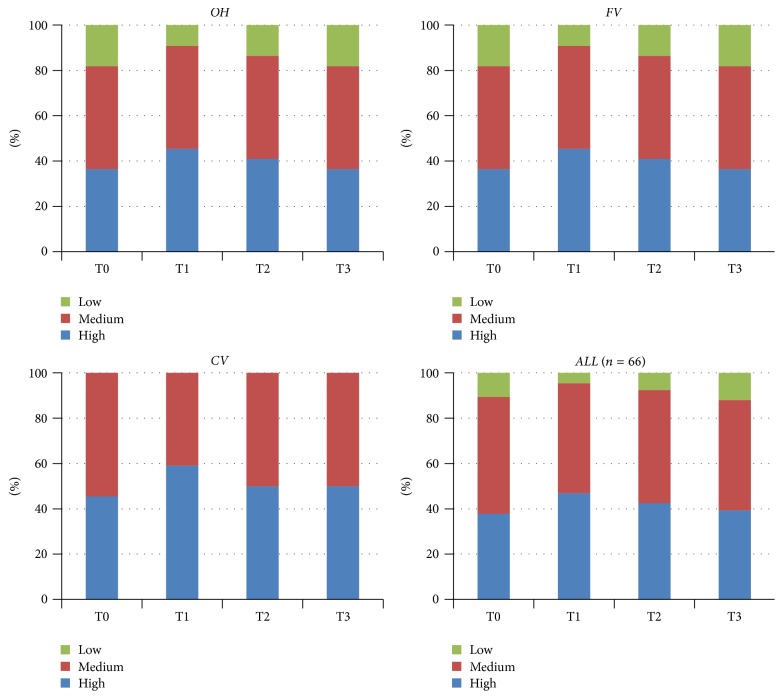
Sample distribution across the different visits (baseline and 1, 4, and 12 weeks) for salivary buffer capacity levels for all groups (*p* values between groups for different visits; T0: 0.161, T1: 0.196, T2: 0.224, and T3: 0.115).

**Figure 4:**
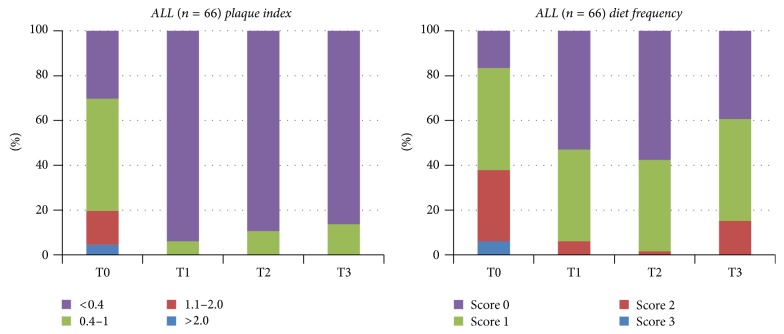
Sample distribution across the different visits (baseline and 1, 4, and 12 weeks) for plaque index and diet frequency levels for all subjects (plaque index *p* values between groups for different visits; T0: 0.347, T1: 0.531, T2: 0.145, and T3: 0.383; diet frequency *p* values between groups for different visits; T0: 0.579, T1: 0.623, T2: 0.569, and T3: 0.830).

The mean DMFT value was found to be 4.59 ± 3.43 for 246 subjects and 8.57 ± 2.17 for 66 (33 female and 33 male) study participants, respectively. No significant differences were found among the three groups regarding demographic properties (gender, age, *p* > 0.05, Table 1). Changes in salivary MS, LB count, buffer capacity, plaque index, and diet frequency levels across the different visits are shown in Figures 1–4.

The only significant difference was found in the CV group for the MS levels during 12 weeks (*p* < 0.05). No significantly different MS levels were observed between the three groups on any visits (*p* > 0.05), except for T2 (Figure 1). A slight decrease was observed for the LB levels (*p* = 0.051). No significantly different LB levels were found between groups on any visits (*p* > 0.05) (Figure 2). A numerical but not significant change was found in the buffer capacity (*p* = 0.28). No significantly different buffer capacity, plaque index, diet frequency, or saliva secretion rate levels were found between groups on any visits (*p* > 0.05) (Figures 3 and 4). A statistically significant decrease was observed for the plaque index levels and diet frequency during the trial (*p* < 0.001).

Statistical analysis revealed that the use of 1450 ppm F toothpaste, fluoride, and chlorhexidine varnish application resulted in a significant modification of the caries risk profile (Cariogram pie chart), thereby increasing the actual chance of avoiding caries in the future at each visit following T0. Tables 2–6 showed the weight of the different sectors of the Cariogram (diet, bacteria, susceptibility, circumstances, and chance of avoiding caries) among participants according to groups.

No significantly different diet, bacteria, susceptibility, circumstances, chance of avoiding caries, or Cariogram risk levels were found at the four measurement times among the groups (*p* > 0.05). Regarding susceptibility level, a more prominent decrease was seen in the FV group than the OH and CV groups at T1, T2, and T3 (*p* < 0.05). These results were affected by fluoride program scores in Cariogram. The chance of avoiding caries increased significantly (*p* < 0.001) during the study for all subjects in the three groups separately: from 46.55 at T0 to 59.0 at T3 for the OH group, from 42.55 to 67.59 for the FV group, and from 42.09 to 60.27 for the CV group, respectively (Table 6). No significant differences were found among the three groups regarding Cariogram risk levels at each visit (*p* > 0.05, Table 7). The Cariogram risk levels decreased significantly for all groups (*p* < 0.001).

## 4. Discussion

The results of the present study showed that the use of 1450 ppm F toothpaste and one visit for application of fluoride or chlorhexidine varnish decreased the subject's caries risk. A significant change in the caries risk as assessed by the Cariogram software was observed for all groups, including high caries risk subjects, during the 12-week trial. No significant differences were found between preventive methods regarding chance of avoiding caries.

Reports about oral health in young adults are rare; thus this study is the first one carried out in Turkey to include high caries risk participants. Young adults are subjected to many changes in life. Leaving school, getting a job, or leaving home to live independently can result in significant lifestyle changes that impact on diet or oral hygiene practices. The lack of proper main meals may result in frequent hunger and a “grazing” eating pattern where an individual eats small amounts of a variety of food all day long. This eating pattern often does not leave enough time for teeth to recover from acid attack and for remineralization to occur. Altered oral hygiene practices often result in lowered use of toothpaste and diminished exposure to fluoride, and this can result in an increase in a subject's caries risk. Therefore, we focused on young adults in this prospective project.

The SiC was used for selection of high caries risk subjects in the present study. Based on these reason, a new index called the SiC was recently introduced to draw attention to those subjects with the highest scores in each population [14]. The SiC is calculated by taking the mean DMFT of the one-third of the individuals with the highest DMFT values in a given population [14]. The fact that there is evidence that past caries experience is the single best predictor for future caries development seems to have been adopted by most clinicians, who appear to more or less ignore multifactorial models. However, main disadvantage of SiC index is that this index is just an extension of DMF index as it follows same criteria for assessing dental caries and will have same limitations in assessing caries in a population as DMF index. Also this index is more of significance in population where caries prevalence is low and has a skewed distribution [15, 16].

It is also important to provide better oral hygiene features by regular tooth brushing and flossing at home, particularly for the high caries risk patients. Therefore, in addition to regularly tooth brushing, application of fluoride or chlorhexidine varnish formulations (with respect to its usage indications) and monitoring in three months recall periods could be considered as an average interval follow-up period for subjects in our study. Many studies have reported that a 12-week time-frame is sufficiently long to enable the semiquantifiable detection of preventive methods. Conversely, other studies suggest that a period of at least six months is preferable in order to detect any adverse or beneficial effects of caries preventive strategies. This study was limited to three months because of concerns that the high caries risk young adults, who were not familiar to regular dental examinations, would not comply with longer term follow-ups for personal and/or socioeconomic reasons.

The only study conducted by combining the evaluation of the activity of preventive methods and the Cariogram program was performed by Mannaa et al., who evaluated the effectiveness of using 5000 ppm fluoride toothpaste using Cariogram for six weeks [11]. In our study, after three months the values regarding all the sections constituting the Cariogram are detected to have decreased significantly in all groups when compared to the baseline. Mannaa et al. stated in their studies that there was a significant decrease in all of the sections after six weeks except for the diet section. Mannaa et al. used only LB level as a base out of the factors belonging to the diet section [11]. In our study, as the change in diet intake frequency of the individuals in addition to the LB level was also followed for three months, this factor may have affected the significant decrease in the diet section.

The effects of the 1450 ppm F toothpaste, fluoride varnish, and chlorhexidine varnish applications that we used in our study on decreasing caries risk have been examined many times and found effective [17–27]. Fluoride is thought to prevent acid production and bacterial growth by inhibiting bacterial metabolism. Fluoride is used in the form of toothpastes, mouthwashes, gels, tablets, varnishes, or pastilles [19]. Activity of fluoride toothpastes is based on fluoride concentration, usage frequency, amount of paste used, and habit of mouth washing after brushing [20]. According to the result of a research conducted on children, caries risk is reported to have decreased significantly with a 4.5-year program that comprises brushing with toothpaste containing 1000 ppm fluoride two times a day [24]. On the other hand, individuals with high caries risk are required to use toothpastes containing 5000 ppm fluoride, which has been accepted recently [11]. However, it is known that topically administered fluoride compounds form CaF_2_ accumulation on the enamel surface. Fluoride varnishes developed for this purpose remain on the tooth surface for a longer period and slowly release fluoride to the oral environment [25]. Fluoride varnishes are recommended to be applied quarterly on individuals in the high caries risk group and once every six months on individuals in the low-risk group [22]. In the “Cariogram,” the fluoride program was set at “2” (fluoride toothpaste, no supplements) at baseline and “1” (additional *F* measures, infrequently) at other weeks. This change in itself changes the weight among the different variables and increases the “actual chance of avoiding caries.” One interesting finding was that the caries risk decreased even when the fluoride exposure was kept at “2” for all four visits. This indicates that the variables influenced by the fluoride varnish regimen accounted for this change in caries risk.

In recent years, chlorhexidine varnishes that are released over the long term and are effective on mouth bacteria have been developed [23]. It is reported in studies conducted on chlorhexidine in individuals with high caries risk that it can suppress MS for a long period and slow down the formation of caries [26]. It is reported that chlorhexidine is released from dentin surfaces treated with chlorhexidine varnish for weeks [27].

We are of the opinion that caries risk assessment methods must be specific to the individual in order to increase the effectiveness of caries management. It is known that detection of an individual's caries risk and active risk factors will facilitate determining the proper preventive methods. For instance, improving only the oral care habits in individuals who have a diet rich in carbohydrates is not adequate; the dietary intake of these individuals should be regulated as well. Applying fluoride gel, mouthwash, or varnish in addition to the toothpaste to an individual with low caries risk symptoms, however, may be unnecessary. Therefore, in our study, before forming the preventive application groups, detailed information regarding diet regulation and application of oral care habits was given to all participants, and each individual was provided with motivation at each appointment.

This study evaluates prophylactic methods therapy important for public health but has some limitations; for example, even if statistically significant differences were obtained, the small number of subjects enrolled in this study might be seen as a shortcoming. Brushing depends on patient cooperation and lack of cooperation might have occurred. Another point to consider is Cariogram software trustworthiness. Taking into account the fact that the software expresses to what extent different etiological factors of caries may affect the caries risk for patient, the variable [fluoride program] influence on caries risk is already established on the costuming settings of the program; therefore, the results can be biased.

It is thought that the habit of brushing the teeth regularly and properly and the interdental plaque removal techniques must first be established, and regular implementation of the proper additional preventive applications in accordance with the individual's specific etiological caries risk factors is important for protection from caries and for reducing the individual to the middle or low caries risk group. The use of short interval control and application of caries risk tests, which motivated the patient about oral care, is one of the most important conclusions to be drawn from our study. The Cariogram program was effective and has some advantages such as making recommendation for preventive care and increasing patient motivation with its pie chart presentation so it can be used in the caries risk assessment instead of single variables.

## 5. Conclusions

The regular and effective short-term (three months) use of 1450 ppm fluoridated toothpaste, one visit application of fluoride, and chlorhexidine varnishes were effective for reducing caries risk in young adults, which can be clearly demonstrated using Cariogram software.

## Figures and Tables

**Table 1:** Demographic variables (*n* = 22).

Groups	Gender: male/female	%	Age (mean ± SD)	(Min–max)
OH	9/13	(41.0/59.0)	21.27 ± 1.24	(20–24)
FV	10/12	(45.0/55.0)	20.59 ± 1.53	(18–23)
CV	14/8	(63.0/37.0)	19.81 ± 1.73	(18–25)

No significant differences were found between the groups regarding demographic variables (*p* = 0,674).

**Table 2:** Diet section results from Cariogram programmes for test groups.

Groups	Baseline	1 week	4 weeks	12 weeks	*p* value^a^
OH	15.86 ± 8.83	10.64 ± 6.74	8.50 ± 3.56	11.18 ± 6.69	<0.001
FV	16.18 ± 7.29	7.14 ± 4.85	7.59 ± 5.05	9.64 ± 5.09	<0.001
CV	17.86 ± 10.07	9.23 ± 3.99	9.82 ± 4.40	11.55 ± 6.40	<0.001
*p* value^b^	0.826	0.096	0.259	0.506	

^a^Comparison within group;  ^b^comparison between groups.

**Table 3:** Bacteria section results from Cariogram programmes for test groups.

Groups	Baseline	1 week	4 weeks	12 weeks	*p* value^a^
OH	13,91 ± 6,63	7,09 ± 4,47	6,55 ± 4,71	8,18 ± 3,91	<0.001
FV	14,27 ± 9,04	4,95 ± 4,06	5,50 ± 4,48	6,91 ± 3,50	<0.001
CV	15,05 ± 7,37	4,05 ± 1,70	5,00 ± 3,98	6,95 ± 4,41	<0.001
*p* value^b^	0,845	0,049	0,263	0,399	

^a^Comparison within group;  ^b^comparison between groups.

**Table 4:** Susceptibility section results from Cariogram programmes for test groups.

Groups	Baseline	1 week	4 weeks	12 weeks	*p* value^a^
OH	18,27 ± 7,00	15,50 ± 7,43	14,59 ± 7,63	16,68 ± 7,86	<0.05
FV	21,23 ± 16,13	9,45 ± 10,01	9,68 ± 9,70	11,55 ± 12,99	<0.001
CV	19,50 ± 9,61	14,09 ± 6,93	15,64 ± 7,11	16,05 ± 7,98	<0.001
*p* value^b^	0,939	0,002	0,003	0,008	

^a^Comparison within group;  ^b^comparison between groups.

**Table 5:** Circumstances section results from Cariogram programmes for test groups.

Groups	Baseline	1 week	4 weeks	12 weeks	*p* value^a^
OH	5,50 ± 3,58	5,32 ± 3,31	4,41 ± 3,26	5,05 ± 3,21	>0.05
FV	5,86 ± 3,01	3,82 ± 2,50	4,00 ± 2,54	4,27 ± 2,43	<0.05
CV	5,50 ± 3,02	5,00 ± 2,99	4,77 ± 2,86	4,95 ± 2,98	<0.05
*p* value^b^	0,902	0,239	0,596	0,778	

^a^Comparison within group;  ^b^comparison between groups.

**Table 6:** Chance of avoiding caries (Cariogram) results from different groups.

Groups	Baseline	1 week	4 weeks	12 weeks	*p* value^a^
OH	46,55 ± 18,86	61,91 ± 16,14	65,91 ± 15,06	59,00 ± 16,51	<0.001
FV	42,55 ± 22,37	74,82 ± 15,22	73,18 ± 16,77	67,59 ± 17,10	<0.001
CV	42,09 ± 19,98	67,59 ± 11,16	65,23 ± 13,93	60,27 ± 15,45	<0.001
*p* value^b^	0,730	0,015	0,169	0,180	

^a^Comparison within group;  ^b^comparison between groups.

**Table 7:** Change of Cariogram risk level results from different groups (H: % 0–30, M: % 31–60, and L: % 61–100).

Groups	Baseline			1 week			4 weeks			12 weeks			*p* value^a^
	H	M	L	H	M	L	H	M	L	H	M	L	
OH	6	11	5	0	11	11	0	8	14	0	11	11	<0.001
FV	5	13	4	0	4	18	0	3	19	1	3	18	<0.001
CV	6	11	5	0	7	15	0	6	16	0	9	13	<0.001
*p* value^b^	1,000			0,204			0,494			0,562			

^a^Comparison within group;  ^b^comparison between groups.
